# Interventions in Wnt signaling as a novel therapeutic approach to improve myocardial infarct healing

**DOI:** 10.1186/1755-1536-5-16

**Published:** 2012-09-11

**Authors:** Kevin CM Hermans, Evangelos P Daskalopoulos, W Matthijs Blankesteijn

**Affiliations:** 1 Department of Pharmacology, Cardiovascular Research Institute Maastricht, Maastricht University, 50 Universiteitssingel, 6229ER Maastricht, PO Box 616 6200MD, Maastricht, The Netherlands

**Keywords:** Myocardial infarction, Wnt, Frizzled, Myofibroblast, Neovascularization, Stem cells, Wound healing, Cardiac remodeling

## Abstract

Following myocardial infarction, wound healing takes place in the infarct area where the non-viable cardiac tissue is replaced by a scar. Inadequate wound healing or insufficient maintenance of the extracellular matrix in the scar can lead to excessive dilatation of the ventricles, one of the hallmarks of congestive heart failure. Therefore, it is important to better understand the wound-healing process in the heart and to develop new therapeutic agents that target the infarct area in order to maintain an adequate cardiac function. One of these potential novel therapeutic targets is Wnt signaling. Wnt signaling plays an important role in embryonic myocardial development but in the adult heart the pathway is thought to be silent. However, there is increasing evidence that components of the Wnt pathway are re-expressed during cardiac repair, implying a regulatory role. Recently, several studies have been published where the effect of interventions in Wnt signaling on infarct healing has been studied. In this review, we will summarize the results of these studies and discuss the effects of these interventions on the different cell types that are involved in the wound healing process.

## Review

### Introduction

Cardiovascular diseases (CVDs) are the leading causes of mortality worldwide. More people die from CVDs than from any other disease. According to WHO, 17.3 million people died from CVDs in 2008, representing 30% of all global deaths. Of these deaths, 7.3 million were due to coronary heart disease [1].

Myocardial infarction (MI) is the result of an occlusion of a coronary artery, caused by a thrombus or an atheromatous ruptured plaque, which deprives the myocardium from sufficient blood flow. This leads to ischemia and eventually death of cardiomyocytes [2]. The location and duration of the occlusion is determinant for the outcome. However, during the last decades, more patients enter the wound-healing phase due to improvements in antithrombolytic and antiarrhythmic therapy.

#### Wound healing after MI

Wound healing is a complex process of consecutive cascades involving several cell types, and finalizes with scar formation. The first phase begins when necrotic cardiomyocytes trigger an inflammatory response by activation of the complement cascade [3]. In humans, the primary inflammatory phase occurs within 12 to 16 hours after MI and mainly attracts polymorphonuclear leukocytes (PMNLs) into the infarcted area. Peak numbers of PMNLs are observed at 24 to 48 hours after infarction. These cells partly remove the cellular debris by phagocytosis and attract lymphocytes, which in turn causes infiltration of macrophages that help to remove the dead cardiomyocytes [2,4]. The infiltrated inflammatory cells not only clear dead cells by phagocytosis but also release proteolytic enzymes and reactive oxygen species, which lead to additional cardiomyocyte death [5]. The second phase is defined by deposition of granulation tissue and starts two to three days after infarction. Here, new extracellular matrix (ECM) proteins are being deposited, starting from the border zone, and progressing into the central area of the infarct later on. Anti-inflammatory cytokines such as transforming growth factor (TGF)-β1, released by a variety of cells [6], play an important role in the initiation of this phase. TGF-β1 triggers the differentiation of fibroblasts into myofibroblasts [7]. These myofibroblasts contribute to the preservation of the structure and morphology of the infarcted tissue via their contractile properties and production of interstitial collagens, which provide tensile strength in the infarcted myocardium [8]. In addition to ECM formation, new blood vessels appear in the infarct three to four days after the ischemic event to supply the injured area with blood. These vessels are derived from pre-existing collaterals or are newly formed from endothelial cells that migrate into the wound [9]. Two to three weeks post-MI (third phase), the infarcted tissue contains (partly) cross-linked interstitial collagens, macrophages, blood vessels and (myo)fibroblasts. Following this, the fourth and final phase is initiated. Granulation tissue matures into a stabilized scar, cells disappear from the wound and most of the collagen becomes cross-linked. Initially, the high ECM content of the scar preserves the ventricular morphology but, when not properly maintained, it eventually loses its coherence resulting in adverse ventricular remodeling [10]. This adverse remodeling involves both the infarcted and non-infarcted myocardium and causes an increased collagen deposition in the interstitial space in the remote areas [11]. There is an increasing body of experimental evidence suggesting that myofibroblasts are responsible for the maintenance of the ECM in the scar [12].

Many cell types contribute to the process of infarct healing, having either a beneficial or detrimental role. Adverse remodeling of the infarct scar will ultimately result in congestive heart failure (CHF). Current therapies are only focused on the delay of the progression of CHF. For this reason, it is important to improve the healing of the scar in such a way that CHF development can be prevented. In recent years, more literature has emerged about Wnt/Frizzled signaling in infarct healing. In this review we will discuss the involvement of Wnt/Frizzled signaling in the different phases of infarct healing and possible therapeutic targets following MI.

### Wnt/Frizzled signaling pathway

The Wnt/Frizzled signaling pathway is a transduction cascade of high complexity, which is fundamental for a wide range of physiological and developmental mechanisms, as well as disease states [13,14]. The involvement of Wnt signaling in the embryonic myocardial development has recently been described in several reviews [15-17]. A large number of different Wnt ligands and Frizzled receptors have been detected in the mesoderm of the heart-forming fields during embryogenesis, implicating their influence on different aspects of cardiogenesis. Under normal physiological conditions the activity of this pathway is significantly reduced and may even be silent in some tissues. However, a general observation in cardiovascular pathology is the re-expression of fetal genes [18]. In several studies, the upregulation of Wnt/Frizzled signaling components was observed after MI, implicating their involvement in cardiac repair [19-23]. Recent evidence indicates upregulated Wnt signaling following MI in progenitor cells, endothelial cells, leukocytes and fibroblasts throughout the entire myocardium [19], suggesting a broad role for Wnt signaling in cardiac repair.

The Wnt/Frizzled signaling pathway comprises of two major branches, the canonical (involving β-catenin) and the non-canonical pathways (independent of β-catenin), which share their main components: Wnts (the ligands) and Frizzleds (their corresponding receptors). Wnts are highly conserved glycoproteins (350 to 400 amino acids long) that are found in many organisms [14]. Nineteen Wnt members have been identified so far in humans and are characterized by large cysteine-rich domains [24]. Moreover, the Wnt proteins are extensively palmitoylated, which makes them highly hydrophobic, hence it is extremely difficult to purify them [25]. Wnts bind to their receptors, Frizzleds, which are seven-transmembrane receptors with sizes varying from 500 to 700 amino acids. Up to the present time, 10 different Frizzled receptors have been identified in mammals and all seem to possess a cysteine-rich domain, which might play a role as a recognition site for the ligand (Wnt) [13,26]. Furthermore, the low-density lipoprotein receptor-related protein (LRP) co-receptors also seem to play an important role in the Wnt/Frizzled signaling cascade. The major members of the LRP family are LRP5 and LRP6 and they act as docking sites for Axin [27].

#### Endogenous Wnt modulators

Modulation of Wnt signaling can occur through the action of several endogenous-secreted proteins. There are two distinct classes, the soluble frizzled-related protein (sFRP) class and the Dickkopf (DKK) class, which function in distinct manners. sFRPs modify Wnt/Frizzled signaling by scavenging Wnt proteins with a frizzled-like cysteine-rich domain and thereby intervene in the interaction between Wnt ligands and Frizzled receptors. The sFRP family also includes the Wnt inhibitory factor 1 (WIF-1) and Cerberus that function in similar ways. DKK proteins inhibit Wnt signaling by interacting with the LRP co-receptors. This causes internalization of the LRPs and thereby diminishing of the availability of co-receptors for Wnt signaling [28,29].

#### The canonical pathway

**Figure 1:**
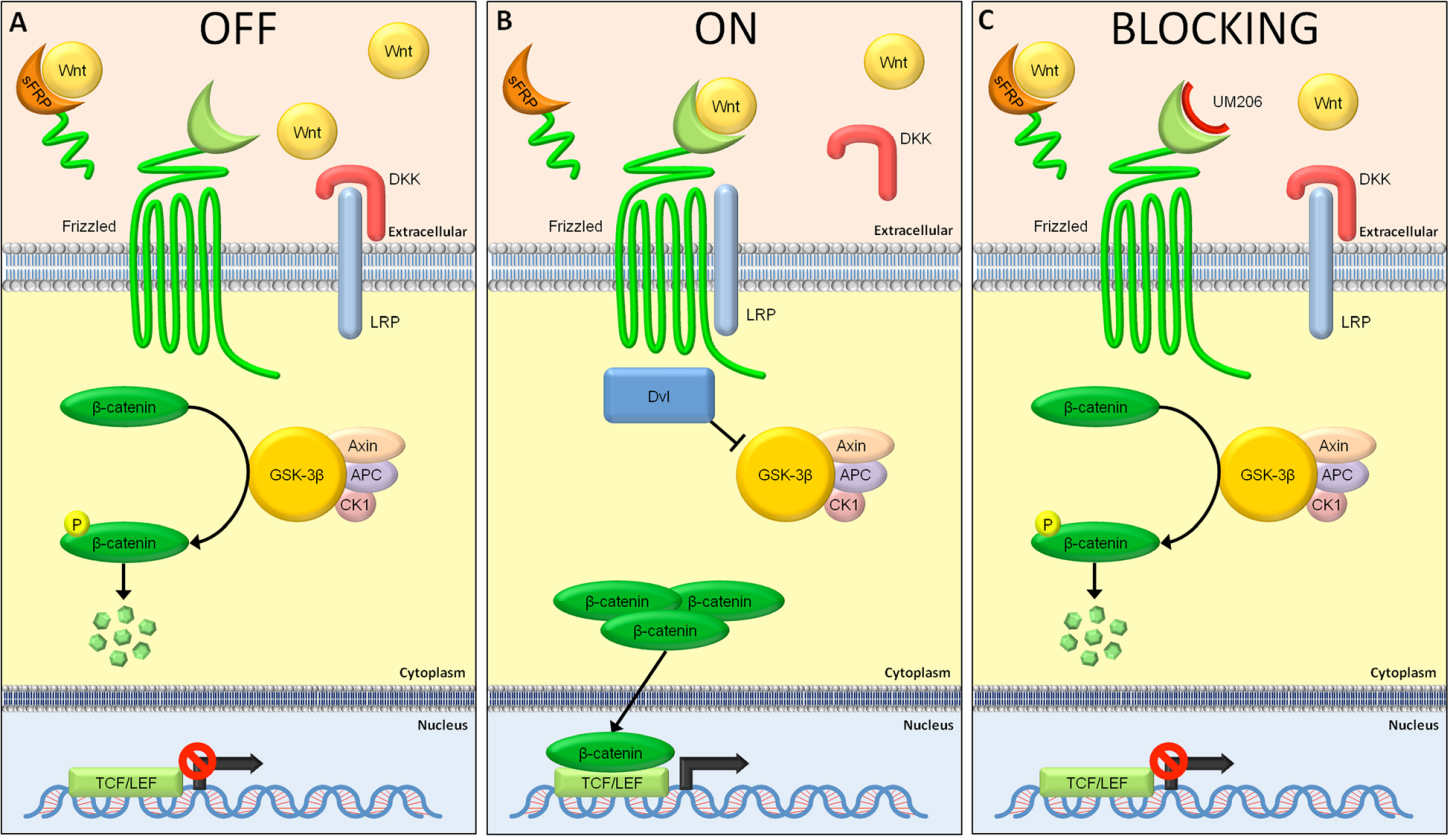
**Different statuses of the Wnt/Frizzled signaling cascade.** (**A**) Wnt/Frizzled signaling is off when the Frizzled receptor is not engaged by a Wnt protein. Hence, the β-catenin degradation complex consisting of GSK-3β, Axin, APC and CK1 phosphorylates β-catenin at Ser/Thr residues, which ubiquitinates the latter and leads to degradation of it. As a result, β-catenin cannot enter the nucleus and activate transcription of target genes. (**B**) Upon binding of Wnt to the Frizzled receptor and co-localization of the LRP receptor, Dvl is activated, which disrupts the destruction complex. Now, β-catenin is accumulating in the cytoplasm and can enter the nucleus where it activates the TCF/LEF proteins and thereby activates the transcription of a broad range of genes. (**C**) Wnt proteins are prevented from binding to the Frizzled receptor by blockade of the cysteine-rich binding domains by UM206. In addition, the endogenous antagonist DKK prevents the LRP co-receptor from co-localization with the Frizzled receptor, thereby blocking the signaling transduction. sFRPs can scavenge Wnt proteins, which can reduce active Wnt signaling. Via all these mechanisms, β-catenin is prevented from entering the nucleus and transcription is not initiated.

β-Catenin is the second messenger of the canonical Wnt signaling pathway and it is the most studied of all. In the absence of the ligand (Wnt), several factors including the adenomatous polyposis coli (APC), casein kinase 1 (CK1) and Axin activate glycogen synthase kinase (GSK) 3β. This in turn phosphorylates several Ser/Thr residues of β-catenin, which leads to the ubiquitination of the latter. Hence, β-catenin is degraded and cannot enter the nucleus in order to modulate gene transcription (Figure1A). On the other hand, upon activation of the Frizzled receptor and the LRP co-receptor by Wnt, the complex (Wnt-Frizzled-LRP) directly activates the first downstream protein, Dishevelled (Dvl). Dvl directly interacts with Axin and breaks down the ‘destruction complex’ comprising of APC, Axin, CK1 and GSK-3β. In this case, β-catenin is no longer degraded but it accumulates in the cytoplasm and enters the nucleus where it binds and activates the T-cell factor/lymphoid enhancer factor (TCF/LEF) proteins. These proteins can bind to DNA and activate the transcription of a wide range of genes, including c-myc, cyclin D1, c-jun, fra-1 [30] and many others (Figure1B). The activation (or not) of these target genes is of paramount importance, since they regulate: cell growth and apoptosis (c-myc) [31], cell proliferation, differentiation and response to various stimuli (c-jun) [32], cell motility and invasion (fra-1) [33] and cell cycle (cyclin D1) [34].

#### The non-canonical pathway

The Wnt/Frizzled transduction cascade is able to signal via mechanisms that do not involve β-catenin. This can be accomplished via the Ca^2+^-dependent pathway, the Ryk/WIF pathway and the Ror2/c-jun N-terminal kinase (JNK) pathway. For a more detailed description of these signaling pathways we refer to some competent reviews on this subject [35,36].

### Myofibroblasts in the infarcted area

The ECM in the heart forms a three-dimensional structure, which serves as a framework for the attachment of myocytes in order to transmit the mechanical force. The cardiac fibroblast is the main cell type that is responsible for producing ECM components. By number, but not by volume, cardiac fibroblasts are the predominant cell type in the heart [37]. In the normal adult heart, collagen half-life is approximately 120 days [8]. For this purpose, cardiac fibroblasts have to be continuously present to replete the degraded ECM components. Cardiac fibroblasts lack a cell-specific marker, hence their staining and histological study is not an easy task [38]. Resting fibroblasts can, under certain circumstances (for example following MI), become activated and exhibit contractile properties due to expression of α-smooth muscle actin (α-SMA) [39]. When a fibroblast undergoes this transition, it differentiates into a myofibroblast. Differentiated fibroblasts also have an increased secretion of profibrotic molecules such as fibronectin and collagen [40,41]. Myofibroblasts are normally not present in the healthy adult myocardium but are abundant under pathological conditions, such as a MI where a fibrotic scar is formed [42]. In addition, dilatation of the infarct is counteracted by the presence of myofibroblasts that produce ECM components and have contractile properties and thereby can confine the development of CHF [12].

#### Origin of myofibroblasts in the remodeling heart

Traditionally, it was thought that myofibroblasts were only derived from resident cardiac fibroblasts. However, myofibroblasts can also be derived from other precursor cells [43-49]. There is evidence that myofibroblasts in the remodeling area of the heart can also originate from endothelial to mesenchymal transition (endo-MT), epithelial to mesenchymal transition (EMT) and circulating hematopoietic bone marrow (BM)-derived cells, also referred to as fibrocytes. Recently, it was shown in mice that a pool of myofibroblasts in the infarct myocardium originated from the epicardium seven days post-MI, suggesting an epicardial EMT process [43]. Endo-MT has been studied in a mouse model of aortic banding, which resulted in a fibrotic heart. It was reported that fibroblasts originating from the endothelium were present in the fibrotic hearts, whereas they were not observed in the unbanded hearts. In the same study, the contribution of BM-derived cells as source of fibroblasts was investigated. It was found that 21.1% of the α-SMA^+^ cells originated from BM cells compared to 3.4% in unbanded hearts [44]. Another study confirms the presence of BM-derived myofibroblasts in a mouse model of MI. Of all myofibroblasts present in the infarct area, 24% originated from BM-cells and were also expressing collagen I [45], whereas in a similar study 57% of the myofibroblasts had a BM origin [46]. More profound research showed that monocytes could be the precursors of the hematopoietic BM-derived myofibroblasts in a MI as well as a pressure overload model [47-49].

#### Role of Wnt/Frizzled signaling in myofibroblasts following MI

There is ample evidence suggesting that components of the Wnt/Frizzled pathway are upregulated in myofibroblasts following MI. Frizzled-2 (Fzd-2) and Dvl-1 have an increased expression four days after MI and this is located at the border zone of the infarct. In the course of time, this expression migrates toward the center of the infarct [22,50]. Previous work from our laboratory suggested that, because of the expression pattern of α-SMA resembles that of Fzd-2, myofibroblasts are the cells in which Wnt signaling is activated [50]. Active canonical Wnt signaling in myofibroblasts following MI in mice was observed by Aisagbonhi *et al*. [21]. *In vitro* experiments done by Carthy *et al*. also showed canonical signaling upon stimulation with Wnt3a in mouse fibroblasts [51]. Wnt3a inhibited fibroblast proliferation but increased migration, α-SMA and TGF-β expression and SMAD2 phosphorylation. Moreover, α-SMA expression was dependent on β-catenin and TGF-β expression [51]. In addition, overexpression of β-catenin also resulted in increased α-SMA expression in cardiac fibroblasts [52], which can be expected since cytoplasmic β-catenin levels are increased when canonical signaling is active (Figure1B). Recently, a cell line of immortalized cardiac fibroblasts was developed in our laboratory. Activation of Wnt/Frizzled signaling with Wnt3a on Fzd-1 transfected fibroblasts increased α-SMA expression, whereas stimulation with Wnt5a decreased α-SMA expression [53]. On the other hand, transfection with Fzd-2 instead of Fzd-1 decreased α-SMA expression upon stimulation with Wnt3a, whereas Wnt5a increased the expression. The same pattern was found for other markers such as collagens and fibronectin [53]. In the same study, overexpression of β-catenin activated β-catenin-mediated signaling and induced overexpression of these markers as well [53]. Additionally, an important finding was the attenuated migration by all tested Wnt/Frizzled combinations [53], which is in contrast to the findings of Carthy *et al*. [51]. On the other hand, proliferation was not affected by any of the combinations [53]. Research on the effect of sFRP-2 on proliferation and differentiation of fibroblasts revealed that sFRP-2 stimulated proliferation but not differentiation, since α-SMA expression was not altered [54].

Taken together, these findings support the hypothesis that differentiation of cardiac fibroblasts and the migrating properties of the myofibroblast can be modulated by Wnt/Frizzled signaling. However, it also emphasizes the complicacy of Wnt/Frizzled signaling in the induction of myofibroblast differentiation.

### Interventions in Wnt/Frizzled signaling following MI

#### Secreted frizzled-related proteins

Barandon *et al*. have extensively studied the role of FrzA (also known as sFRP-1) in cardiac repair [23,55,56]. In their first study, transgenic (Tg) mice overexpressing FrzA were subjected to MI. FrzA overexpression had a profound effect on the healing process following MI [23]. The incidence of rupture and the size of the infarct area were decreased and cardiac function was improved in FrzA Tg mice. The cellularity in the scar was improved and was mostly composed of myofibroblasts, confirmed by α-SMA staining. Moreover, myofibroblasts and collagen deposition were more concentrically aligned to the endo- and epicardium in transgenic mice. Also matrix metalloproteinase (MMP)-2 and −9 activity was reduced [23], which decreases the chance of cardiac rupture [57,58]. In addition, early leukocyte infiltration as well as the apoptotic index was decreased in the first week after MI [23].

Subsequently, the role of FrzA on angiogenesis following MI was assessed. The capillary density in the scar was significantly higher in the FrzA Tg mice. Moreover, the vessel walls were more muscularized and the mean vessel lumen area was 3-fold higher compared to wild-type controls [55]. Further investigation into the role of FrzA and the involvement of Wnt/Frizzled signaling in the post-ischemic inflammatory process elucidated that FrzA overexpression in leukocytes altered the inflammatory response following MI [56]. Neutrophil infiltration was significantly reduced for up to seven days after MI, but there was no difference in T-lymphocyte or macrophage infiltration. The pro- and anti-inflammatory cytokine profile changed as well, since FrzA significantly reduced interleukin-6 and increased interleukin-10 expression. This correlated with a reduction in cardiac rupture, scar size and an overall improvement in cardiac function [56]. Modifications in inflammatory responses by Wnt signaling have also been confirmed in other studies that indicate that Wnt signaling is pro-inflammatory [59-61].

sFRP-2 has also been shown to play a role in myocardial infarct healing [54,62]. *In vitro*, recombinant sFRP-2 was shown to inhibit the procollagenase activity of bone morphogenic protein (BMP)-1 in primary cardiac fibroblasts, preventing the maturation of type I pro-collagen [54]. Following MI, newly synthesized type I and type III collagens were strongly upregulated in the infarct area at day 3 and expression levels remained high thereafter. Endogenous sFRP-2 was upregulated in the infarct area and peaked after three days, followed by a decrement to undetectable levels after fourteen days. The same pattern was observed for BMP-1 [54]. Recombinant sFRP-2 injection directly into the heart, two days after MI, reduced fibrosis by approximately 66%. Even one month after induction of MI, when the remodeling phase is normally complete, the amount of fibrosis was still reduced by approximately 50% [54]. This confirms that sFRP-2 inhibits collagen maturation in the scar and thereby has a distinct effect on this process compared to sFRP-1 [23]. In addition, four weeks after MI, the ratio of the anterior to posterior wall thickness decreased significantly in control animals, whereas this was not the case in sFRP-2 treated animals. This resulted in an improved cardiac function four weeks after treatment since fractional shortening (FS) was increased [54].

In contrast to these data, Kobayashi *et al*. demonstrated contradictory results for sFRP-2 [62]. It was found that sFRP-2 has the ability to enhance the activity of BMP-1 instead of inhibiting it. This was confirmed in fibroblast cell cultures of sFRP-2 null mice, where reduced pro-collagen processing, and deposition of collagen and ECM were observed [62]. Following MI in mice there was an upregulation of sFRP-2 mRNA expression at day 4, which peaked at day 7. At day 14 it was reduced but there was still an increase of 30 to 40-fold. However, an induced expression on protein level was not demonstrated. In infarcted sFRP-2 null mice, Sirius red stained cross-sections showed only 15 to 20% fibrosis of the left ventricle (LV), compared to 25 to 35% in wild-type mice. Also cardiac function was preserved as the ejection fraction (EF) was significantly improved two weeks post-MI in sFRP-2 null mice [62]. So whereas He *et al*. demonstrated that after MI, sFRP-2 injection in the infarct area improves cardiac function and is able to inhibit fibrosis and remodeling [54], the latter study by Kobayashi *et al*. concluded that the same phenotype could be observed in sFRP-2 null mice [62]. Even though the effect of sFRP-2 is completely opposite in the two studies, Kobayashi *et al*. monitored the infarct only up to two weeks [62]. At this time the wound healing is not completed yet, thus the effect on the complete healing phase is unknown and needs further investigation.

sFRP-4 has been shown to play a profound role in infarct healing as well [63]. In a MI rat model, sFRP-4 mRNA expression in the ischemic area was upregulated. Following MI, it peaked at three to five days to a 4-fold increase, and decreased to baseline levels after a month. Administration of recombinant sFRP-4 in the ischemic regions increased cardiac function in a dose-dependent manner. The size of the LV cavity remained normal whereas this was enlarged in control animals. Also the acellular scarring was suppressed in sFRP-4 treated hearts, resulting in a better infarct healing [63].

Collectively, these studies implicate that sFRPs play an important role in infarct healing and this is probably not only by the modulating effect between Wnt ligands and Frizzled receptors but also by other inhibitory or stimulating properties (for example on BMP-1).

#### Synthetic pharmacological tools

Recently, pyrvinium (an FDA-approved drug) has been described to possess Wnt signaling inhibiting properties [64]. It potentiates the downstream molecule CK1, which is a component of the β-catenin degradation complex. This drug was administered in the peri-infarct area, directly after infarct induction. After 30 days, LV internal diameter in diastole (LVIDD) was significantly decreased compared to control. No other differences in infarct size or morphology were reported. An increased cell proliferation was observed in the border zone and the remote area of the pyrvinium-treated hearts, which suggests that cardiomyocytes re-entered the cell cycle. This was not due to a better vascularization, since there was no difference between the groups, but most probably due to inhibition of Wnt signaling in cardiomyocytes [64]. A limitation of this study was that no collagen levels, MMP expression or other important components for wound healing were checked.

In our laboratory, we have developed a peptide fragment of Wnt5a (UM206) that occupies the binding sites for Wnt3a and Wnt5a on the Fzd-1 and −2 receptor, thereby preventing Wnt signaling (Figure1C) [65]. This peptide showed promising results in a mouse model of MI. Administration of UM206 for five weeks via osmotic mini pumps completely prevented death due to heart failure, whereas approximately 30% of the control animals died after five weeks. Moreover, infarct size was decreased by UM206 treatment, while neovascularization and myofibroblast numbers were increased. Furthermore, total collagen levels in the infarct were decreased, though collagen Iα1 was upregulated whereas collagen III was downregulated [65]. Collagen I has a higher resistance to dilatation of the infarct compared to collagen III and probably thereby contributes to preservation of cardiac morphology [66]. Hence, treatment with UM206 improved cardiac function markers and completely prevented the development of heart failure [65].

In conclusion, these studies provide evidence that inhibition of Wnt/Frizzled signaling can have beneficial effects on wound healing and can prevent heart failure after MI.

#### β-Catenin

β-catenin is a multifunctional protein. It can serve as a membrane protein that links the cytoplasmic tail of cadherin to the actin cytoskeleton by which it maintains the tissue architecture and cell polarity. On the other hand, cytoplasmic β-catenin can act as a co-activator to induce transcription of target genes [67].

Modifications in the expression of β-catenin can also serve as a tool to inhibit or promote Wnt/Frizzled signaling, since it is a downstream component of the cascade. *In vitro* overexpression of β-catenin in cardiomyocytes and cardiac fibroblasts of rat origin resulted in reduced apoptosis. It also enhanced vascular endothelial growth factor (VEGF) expression in both cell types and increased α-SMA expression in fibroblasts. Besides, it promotes cell cycle progression but only cell numbers of cardiac fibroblasts were increased [52]. Based on these results, an *in vivo* study was performed that had promising results [52]. In a rat model of MI, overexpression was achieved by injecting a β-catenin containing adenoviral vector in the infarct area following induction of MI. One week after infarction, the amount of apoptotic cells, as well as the size of the infarcted area, was smaller in the β-catenin transfected group. Also FS was improved compared to control animals. In concordance with the *in vitro* data, there was an increment in VEGF expression and capillary density [52]. These data suggest that β-catenin overexpression is able to reduce infarct size and to improve cardiac function by decreasing apoptosis and increasing capillary density. However, as with the *in vitro* data, no information on α-SMA expression in the infarcted area was reported, which could give an indication for a correlation between cardiac function and the presence of myofibroblasts. Remarkably, another study showed more favorable results with β-catenin depletion instead of overexpression [68]. Here, cardiomyocyte-specific depletion of β-catenin resulted in decreased infarct size and mortality as well as improved FS four weeks after infarction, though there was no difference in apoptosis. Therefore, apoptosis could not explain the improved cardiac phenotype. Further research to certify these effects led to the discovery of enhanced differentiation of cardiac progenitor cells in the infarcted area [68]. This suggests that resident precursor cells contribute to the endogenous regeneration of cardiac tissue in LV remodeling following MI and that this is amplified by downregulation of β-catenin. The latter two studies intervene at different sites in the myocardium (depletion of β-catenin in viable cardiomyocytes versus injection in infarcted area), which may explain the discrepancy. Overexpression of β-catenin simulates the stimulation of canonical Wnt/Frizzled signaling, whereas depletion is associated with inhibition of Wnt/Frizzled signaling, therefore the latter study is in concordance with previous studies that showed beneficial results with inhibition of signaling by means of antagonizing tools.

### Neovascularization following MI and the role of Wnt/Frizzled signaling

Vascularization is important for maintenance of all tissues in the body. The importance of regulated Wnt signaling in vasculogenesis during early developmental stages has been shown in several studies with disruptions or mutations in the Wnt/Frizzled pathway. Deletion of Wnt-2 or Fzd-5, results in defects of the placental vasculature in mice [69,70]. Moreover, Wnt-7b has been shown to be crucial in the development of the pulmonary vasculature [71]. Furthermore, normal expression of Fzd-4 and LRP5 is required for vascular organization during embryogenesis [72]. Vessel formation and remodeling can also be one of the processes during pathological conditions such as wound healing following MI. The implication of Wnt/Frizzled signaling has also been established in such conditions. In an earlier report from our laboratory, it was demonstrated that cytoplasmic β-catenin and Dvl-1 expression were located in the endothelial cells of the newly formed and pre-existing blood vessels of the infarcted area one week post-MI, whereas this was not observed in the rest of the heart [73]. More recently, local administration of DKK-2 in the infarcted heart has been shown to enhance neovascularization [74]. This implies that impairment of Wnt/Frizzled signaling ameliorates formation of new vessels. This concept is strengthened by *in vivo* MI studies, which demonstrate increased amount of blood vessels in the infarcted area when treated with the antagonist UM206 [65] or overexpression of FrzA [55].

Taken together, the involvement of Wnt/Frizzled signaling in formation of new vessels during pathophysiological conditions is highly evident. Modulation in this signaling cascade following MI is a potential means of improving infarct healing.

### The role of Wnt/Frizzled signaling in stem cells following MI

Until the recent discovery of cardiac stem cells (CSCs), the heart has been viewed as a terminally differentiated organ. These cardiac progenitor cells are multipotent and can give rise to new cardiomyocytes, smooth muscle cells and endothelial cells [75,76]. Hence it was hypothesized that these stem cells could be a potential tool for the prevention or cure of heart failure following MI. Recently, isolated c-kit^+^ human CSCs have been shown to differentiate into myocytes, endothelial cells and smooth muscle cells *in vitro*. These differentiated myocytes were able to contract upon electrical stimulation [77]. Injection of human CSCs into the infarcted rat/mouse heart resulted in a chimeric heart that was integrated into the myocardium of the rodent and contributed positively to the cardiac performance [77]. Recently, it was shown that acute MI increased c-kit^+^ and Sca-1^+^ cells in the circulation [78]. These cells were also increased in the BM one week after MI, indicating an activated BM niche. In addition, it was demonstrated that canonical Wnt signaling was activated in mononuclear BM cells of the mice. Furthermore, Wnt signaling activated human BM cells and increased their migratory capacity *ex vivo*[78], implicating a role for Wnt/Frizzled signaling in stem cells. Overexpression of β-catenin in *ex vivo* expanded hematopoietic progenitor cells (HPCs) has been shown to decrease infarct size and improve EF, FS and LV end systolic diameter in mice following injection of these cells [79], whereas cardiomyocyte specific β-catenin depletion enhanced cardiac progenitor cell differentiation and improved the cardiac phenotype [68]. Besides HPCs, mesenchymal stem cells (MSCs) are also easy to isolate from the BM and can be expanded *ex vivo* without losing multipotency [80]. Akt overexpression in MSCs augmented the endogenous expression and release of sFRP-2. The injection of conditioned medium produced from these cells in infarcted hearts has been shown to reduce infarct size [81]. Moreover, injection of sFRP-2 overexpressing MSCs in the border zone of the infarct ameliorated the engraftment and improved the cardiac phenotype following MI [82].

Wnt signaling is necessary for maintenance of pluripotency [83], whereas the Wnt signaling inhibitor DKK-1 is critical for proliferation [84]. Also, sFRP-2 is responsible for MSC self-renewal by inhibition of the BMP and Wnt pathway and may improve cardiac wound healing mediated by MSC engraftment [85]. Furthermore, Wnt3a decreases the proliferation of CSCs by the activation of the insulin-like growth factor binding protein 3 through canonical Wnt signaling. In addition, intramyocardial injection of Wnt3a following MI impedes endogenous cardiac regeneration and deteriorates cardiac function [86]. Taken together, these data show that modification of Wnt/Frizzled signaling can contribute to the actions of stem cells during cardiac repair.

## Conclusion

**Figure 2:**
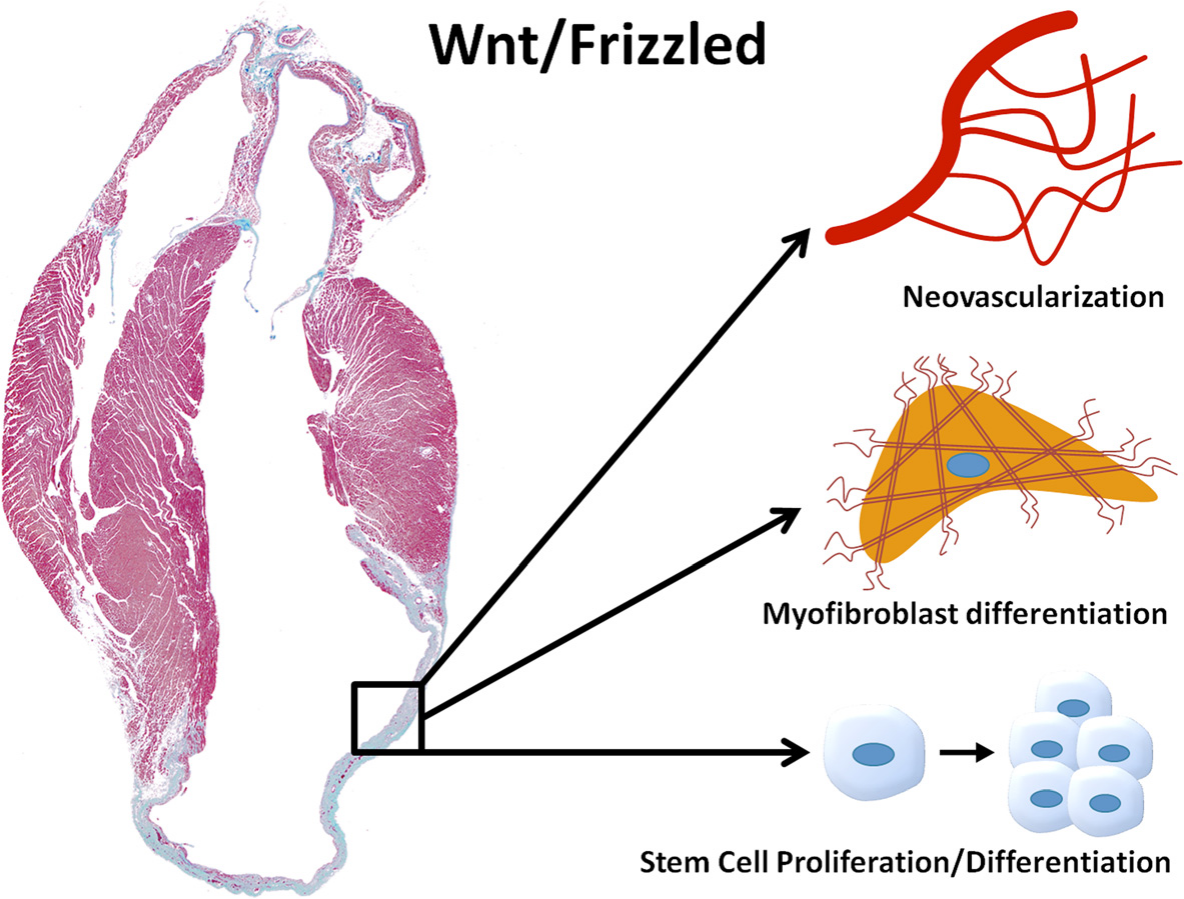
**Potential targets of modulations in Wnt signaling to improve cardiac repair.** Interventions in Wnt/Frizzled signaling can modulate several processes such as neovascularization, myofibroblast differentiation and stem cell proliferation/differentiation, which can all contribute to improved healing of the scar, preservation of cardiac function and thereby prevent development of congestive heart failure (CHF).

The wound-healing phase is a critical process for the clinical outcome of patients who have suffered from MI. Abnormal remodeling will result in a poorly healed scar that can cause dilatation of the left ventricle and ultimately progresses into CHF, which can be lethal. Since an increasing number of patients are affected by this problem, it is of major importance that therapeutic interventions do not just delay the progression, but rather prevent the development of CHF. The Wnt/Frizzled pathway is involved in many aspects of cardiac repair following MI and may be a promising therapeutic site for interventions. However, the precise targets and mechanism of intervention are still a matter of debate. Thus far, promising tools have been used in experimental settings, targeting Wnt/Frizzled signaling at several important sites involved in infarct healing such as (myo)fibroblasts, progenitor cells, neovascularization, inflammation, collagen maturation and several others that can contribute to an improved cardiac phenotype by minimizing the scar and reducing ventricular dilatation (Figure2). Wnt/Frizzled signaling is mostly silent under physiological conditions but its components can be upregulated in a pathological state. Because this upregulation is only taking place in the affected organ, pharmacological tools display a relative selectivity at this site, thereby not affecting other systems or organs. Therefore, development of strategies that modulate Wnt/Frizzled signaling can serve as novel therapeutic tools to improve infarct healing and quality of life in patients that have had a MI.

The discussed studies, in which interventions in Wnt signaling following MI are described (Table1), have different modes of treatment that may explain the discrepancy in the outcomes. Though, there is a trend that improved outcomes are more often achieved by inhibition of Wnt signaling. Therefore, in our opinion, blockade of Wnt/Frizzled signaling would be the best strategy to intervene following MI. In addition, many aspects of the pharmacology and the activation of the different signal transduction pathways by different combinations of Wnts and Fzds are far from completely understood. Therefore, more research is needed in testing different Wnt/Fzd combinations and the subsequent activation of the signaling pathway. Besides, further investigations are necessary to compare interventions in different types of MI (permanent ligation versus ischemia and reperfusion) and different animal models.

**Table 1:** Overview of studies intervening in the Wnt pathway following MI

**Study**	**Intervention**	**Effect on Wnt pathway**	**Outcome**
Barandon *et al*. [23]	FrzA overexpression	Inhibition	Reduced MI size and improved cardiac function
Barandon *et al*. [56]	sFRP-1 overexpression	Inhibition	Decreased cardiac rupture and scar size
He *et al*. [54]	sFRP-2 administration	Inhibition	Improved cardiac function
Kobayashi *et al*. [62]	sFRP-2 deletion	?	Decreased fibrosis and improved cardiac function
Matsushima *et al*. [63]	sFRP-4 i.m. administration	Inhibition	Improved cardiac function
Saraswati *et al*. [64]	Pyrvinium administration	Inhibition	Decreased LVIDD
Laeremans *et al*. [65]	UM206 administration	Inhibition	Decreased infarct size and improved cardiac function
Hahn *et al*. [52]	β-catenin	Stimulation	Decreased infarct size
	overexpression		
Zelarayan *et al*. [68]	Cardiomyocyte specific		
	- β-catenin depletion	- Inhibition	- Improved cardiac function
	- β-catenin stabilization	- Stimulation	- Opposite effects

LVIDD, left ventricular internal diameter in diastole; MI, myocardial infarction; sFRP, soluble frizzled-related protein.

## Abbreviations

α-SMA = α-smooth muscle actin; APC = adenomatous polyposis coli; BM = bone marrow; BMP = bone morphogenetic protein; CHF = congestive heart failure; CK1 = casein kinase 1; CSC = cardiac stem cell; CVD = cardiovascular disease; DKK = Dickkopf; Dvl = Dishevelled; ECM = extracellular matrix; EF = ejection fraction; EMT = epithelial to mesenchymal transition; Endo-MT = endothelial to mesenchymal transition; Fzd-2 = Frizzled-2; FS = fractional shortening; GSK = glycogen synthase kinase; HPC = hematopoietic progenitor cell; JNK = c-jun N-terminal kinase; LRP = low-density lipoprotein receptor-related protein; LV = left ventricle; LVIDD = left ventricular internal diameter in diastole; MCS = mesenchymal stem cell; MI = myocardial infarction; MMP = matrix metalloproteinase; PMNL = polymorphonuclear leukocyte; sFRP = soluble frizzled-related protein; TCF/LEF = T-cell factor/lymphoid enhancer factor; Tg = transgenic; TGF = transforming growth factor; VEGF = vascular endothelial growth factor; WIF = Wnt inhibitory factor.

## Competing interests

The authors declare that they have no competing interests.

## Authors’ contributions

KCMH carried out the bibliographic search, created the figures and wrote the draft. EPD contributed to this. WMB coordinated and helped with the draft of the manuscript. All authors read and approved the final manuscript.
